# Temporal Annotation of German Clinical Language in Real and Synthetic Clinical Documents: Corpus Development and Baseline Tagger Validation Study

**DOI:** 10.2196/71458

**Published:** 2026-02-25

**Authors:** Luise Modersohn, Udo Hahn

**Affiliations:** 1 Chair of Medical Informatics Institute for AI and Informatics in Medicine Technical University of Munich (TUM) university hospital Munich, Bavaria Germany; 2 JULIELab Friedrich Schiller University Jena Jena Germany; 3 Institute for Medical Informatics, Statistics and Epidemiology (IMISE) Leipzig University Leipzig Germany

**Keywords:** natural language processing, clinical NLP, German clinical language, corpus development, temporal named entity, temporal relations, annotation guidelines, annotation process

## Abstract

**Background:**

Temporal information about patients constitutes a precious source for clinical decision-making and medical treatment. The automatic extraction of such data from unstructured clinical narratives requires time-annotated clinical reports and notes from which time-informed taggers can be learned. Unfortunately, the non-English clinical language community, the German one as a typical example, with only a few exceptions, generally lacks such time-annotated resources to train and evaluate temporal taggers.

**Objective:**

To overcome this metadata bottleneck, we developed a TimeML-conformant annotation schema for both temporal entities and temporal relations adapted to the needs of German medical language. Based on the annotations derived therefrom, we trained state-of-the-art baseline taggers to recognize temporal expressions in clinical documents.

**Methods:**

Starting from temporal annotation guidelines for English clinical documents, we developed preliminary annotation guidelines for temporal named entities and temporal relations for the German language. These guidelines were subsequently refined and adapted to German clinical jargon, incorporating the work experience of 5 clinically trained annotators (students of medicine). For this task, we used randomly selected smaller subsets of 2 German clinical corpora—a real-world one (3000PA_J_) and a synthetic one (GraSCCo). Both corpora were annotated (3000PA_J_ partially, GraSCCo completely), randomly selecting 10% of the documents as an agreement part on 3000PA_J_. To measure interannotator agreement (IAA), we computed pairwise *F*_1_-scores. We used that metadata to develop BERT (Bidirectional Encoder Representations from Transformers)-based language models for the creation of time-sensitive baseline taggers. All annotations are based on TimeML, the international de facto standard for time information markup.

**Results:**

We created 3000PA_J_-temp, a time-annotated corpus of real clinical documents (which cannot be distributed because of the rigid privacy legislation enforced for German clinical data), and GraSCCo-temp, a synthetic one (which is publicly available without any restrictions). Based on the final guidelines, we achieved an IAA *F*_1_-score of 0.9 on both corpora for the temporal named entity recognition task. For the temporal relation extraction task, the IAA on GraSCCo plummeted to an *F*_1_-score of 0.57 and 0.41 on 3000PA_J_, respectively. Still, those results are comparable with English clinical datasets. Our baseline tagger for named entities reached *F*_1_-scores between 0.64 and 0.85. For automatic relation extraction, we achieved *F*_1_-scores ranging between 0.60 and 0.64.

**Conclusions:**

We here introduce the first TimeML-compliant annotation scheme for time expressions occurring in German clinical language and apply it to 2 clinical corpora, one with nondistributable real clinical data, the other with distributable synthetic ones. The latter constitutes the first publicly accessible, temporally annotated clinical corpus for the German language. The time tagger trained on these datasets is the first of its kind, fully compliant with the TimeML markup language. The amounts of temporal metadata in our corpora are among the largest datasets ever produced for the clinical domain, both compared with English and German predecessors.

## Introduction

### Overview

**Figure 1:**
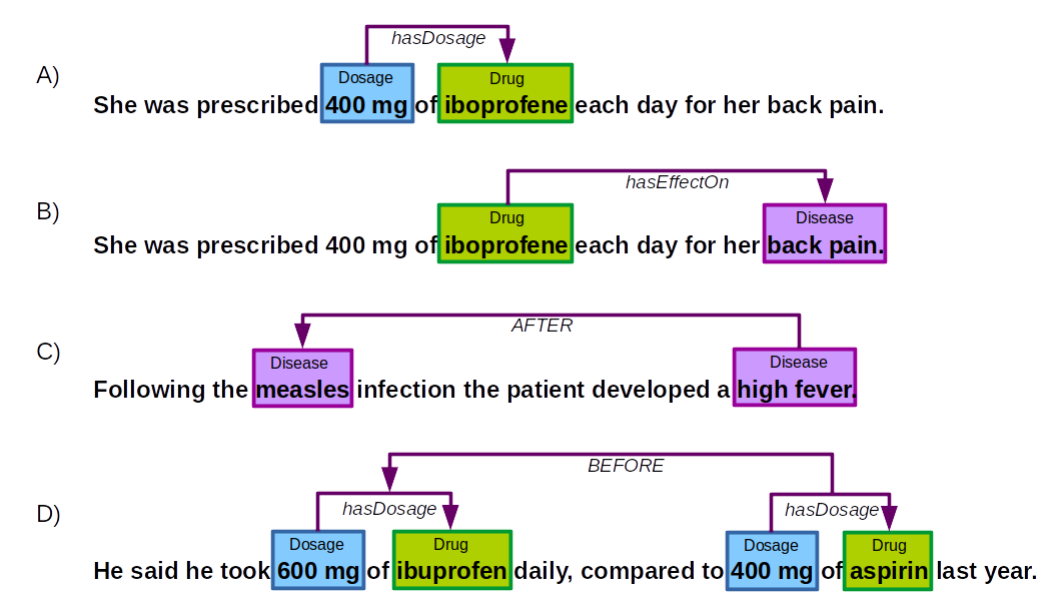
Examples for named entities, semantic, and temporal relations.

Information extraction is a field of natural language processing (NLP) that is concerned with automatically identifying mentions of entities of interest (so-called named entities) and semantic relations holding among them [1-3]. In the medical domain, commonly researched named entity types are, for example, Symptom, Finding, Diagnosis, Disease, or Drug, that are conceptually linked via medically relevant semantic relation types, such as Drug – hasDosage – Dosage, Drug – hasEffectOn – Disease (examples are shown in Figures 1A and 1B). Whereas named entity types address the terminological level of medical knowledge, including its taxonomic structure, semantic relations help represent single pieces of factual knowledge (medical assertions or facts).

Sets of medical entities and facts identified by information extraction tools form a bag of information units lacking further structure. One particularly important dimension to add structure to otherwise unrelated information pieces focuses on their temporal ordering on the time axis, a class of information we here refer to as temporal knowledge [4-7]. Information about the temporal order of (clinical) events is crucial in a hospital setting, because, for example, a drug reported on admission has a medically different status compared with one prescribed at discharge. Ultimately, temporal knowledge can be used to visualize timelines of patients' histories, which might prove helpful for doctors in medical decision making and for nurses in their daily care routines. This type of knowledge can either be encoded in terms of the named entity types “Event” and “Temporal Expressions,” such as Date, Duration, and Time, and “temporal relations,” which impose a (temporal) ordering on named entities or semantic relations. For instance, we may state a temporal relation between named entities, such as AFTER (examples are shown in Figure 1C), or between semantic relations, such as BEFORE (examples are shown in Figure 1D).

Prior to the automatic extraction of named entities, semantic and temporal relations, specialized classifiers (taggers) have to be pretrained or fine-tuned, typically using manually supplied annotations to guide the machine learning process. We here face a serious disparity between corpora for English and non-English languages (such as German, the focus of our work [8]). Based on an extensive survey of temporal datasets and tagging tools in the Related Work section, we encounter a large number of temporally annotated resources for the English language, both for clinical and nonclinical domains, that have no comparable counterpart for the German (clinical and nonclinical) language. For German, such resources are scarce and, if available, rather small in the nonclinical domain, while in the clinical domain, they are almost nonexistent. Moreover, annotations obeying the full TimeML annotation language standard are entirely missing for German.

Accordingly, in the Methods section, the unique contributions of this article are described in depth: (1) the provision of 2 clinical German-language corpora, a nondistributable real clinical corpus (3000PA_J_) and a distributable synthetic corpus (GraSCCo), both of which have been richly annotated, adhering to TimeML, the current meta-language standard for temporal annotations. 3000PA_J_ and GraSCCo are the first corpora annotated with TimeML markup for the German language ever. (2) Exploiting these annotations, we trained the first transformer-based baseline taggers that automatically extract temporal information encoded in German clinical reports and notes. Up until now, transformer-based temporal taggers are not even available for the general German language (eg, covering news or social media content).

### Related Work

Temporal information about patients constitutes a precious source for clinical decision making and medical treatment. It forms cohesive ties between single factual statements by linking them via temporal relations (see the examples for AFTER and BEFORE in Figure 1), which are the basis for temporal reasoning (eg, AFTER (x,y) and AFTER (y,z) transitively imply AFTER (x,z)). The automatic extraction of such temporal data is a prerequisite for the overarching goal to automatically construct consistent, maximally complete, and chronologically coherent timelines of the patients' medical history from unstructured clinical documents and structured electronic medical records to help support medical diagnosis, treatment decisions, and prognosis [9-20].

The automatic extraction of such temporal information from unstructured medical records (clinical reports, notes, or free text fields in the electronic medical record) requires language models trained on raw or metadata-enriched, that is, annotated, textual data. However, annotated temporal data resources are still quite rare and often seriously limited in size. Shortage of corpora with temporal metadata, however, directly hinders supervised learning of time-sensitive language models.

The starting point of time-centered knowledge representation research dates back to Allen’s time algebra [21], which was then adopted in the design of TimeML [22], a specification and annotation language for temporal natural language utterances that combines temporal entities (Dates, Durations, Events, etc) and relations (eg, BEFORE, AFTER, OVERLAP); temporal relations impose temporal orderings on temporal entities. After years of experimentation, TimeML evolved into an ISO (International Organization for Standardization) standard as ISO-TimeML [23]. Part of TimeML, and 2 of its historical predecessors, are TIDES TIMEX2 [24] and TIMEX3 [23], which are used to mark up explicit temporal expressions, such as time expressions, dates, durations, etc. Table 1 illustrates the dominant role of TimeML as the representational foundation (markup or annotation language) for time-annotated corpora.

**Table 1:** Time-focused (mostly English only) nonclinical corpus resources.

Corpus/(challenge) task (year)	Functional characterization of (challenge) task	Annotation language	Text genre	Size in tokens	Type/number of metadata
TimeBank 1.1 [25] (2003)	N/A^a^ (corpus)	TimeML 1.1	186 news articles	68.5k	13 temporal relation types: 5.1k temporal relation annotations; 11.2k annotation items, in total
TimeBank 1.2 [26] (2007); for error correction see the work of Ocal et al [27]	N/A (corpus)	TimeML 1.2	186/183 news articles	68.5k	1.4k TIMEX3 annotations; 7.9k Event annotations; 13 temporal relation types (6.4k temporal relation annotations and 27.6k annotation items, in total)
TimeBank-Dense [28,29] (2014)	N/A (corpus)	Subset of TimeML 1.2	36 news articles (subset of TimeBank 1.2)	13.3k (estimated)	0.3k TIMEX3 annotations; 1.7k Event annotations; 6 temporal relation types (12.7k [10.8k] temporal relation annotations; 14.7k annotation items, in total)
TempEval-1 [30] (2009)	Temporal relation (TLink) identification	Subset of TimeML 1.2 (TIMEX3, Event, TLink)	182 news articles (subset of TimeBank 1.2)	61.4k [31]	6 temporal relation types: 5.8k temporal relation annotations; 13.9k annotation items, in total [28]
TempEval-2 [32] (2010)	TIMEX3 identification; Event identification; temporal relation (TLink) identification	Subset of TimeML 1.2 (TIMEX3, Event, TLink)	Same as TempEval-1	63k	6 temporal relation types: 4.9k temporal relation annotations; 12.7k annotation items, in total (English data only) [28]
TempEval-3 [31] (2013)	TIMEX3 extraction and normalization; Event extraction and classification; end-to-end temporal relation (TLink) identification	Subset of TimeML 1.2 (TIMEX3, Event, TLink)	182 news reports (TimeBank) + 73 news reports(tiny subset of≈1M)(AQUAINT [33]);255 news articles, in total	Gold: 61.4 k (TimeBank) + 6.4 k +34.0 k (tiny subset of AQUAINT [33]) = 101.8k, in total; Silver: 666k (Gigaword [32])	13 temporal relation types (full TimeML set): 11.1k temporal relation annotations; 24.2k annotation items, in total (English data only) [28]
SemEval 2015 - TimeLine Task 4 [34] (2015)	Cross-document analysis of news articles to construct timelines for target events, that is, detecting, anchoring in time, and ordering events for a target entity	Subset (and minor adaptation) of TimeML 1.2	120 WikiNews articles, with 4 main topics: Apple, Airbus & Boeing, General Motors, Chrysler & Ford, stock market	10k per main topic, 40k, in total	0.9k Event annotations;0.7k Event chain annotations; 38 target entities; 37 timelines
RED [35] (2016)	N/A (corpus)	Subset of TimeML	95 news articles and casual discussion forum chats	54.3k tokens	1.1k TIMEX3 annotations; 8.7k Event annotations; 5 TLink types (Before, Overlap, Begins_on, Ends_on, Simultaneous): 1.4k temporal relation annotations (TLinks)
CaTeRS [36] (2016)	N/A (corpus)	Event definition based on the TRIPS ontology; subset of TimeML TLINKs	320 five-sentence short stories	1600 sentences	2.7k Event annotations;4 TLink types (Before, Overlaps, Contains, Identity): approximately 2.2k temporal relation annotations (TLinks) out of 2.7k causal and temporal relations
Event StoryLine (ESC) v0.9 [37] (2017)	N/A (corpus)	Compatible with TimeML	258 news articles about calamityevents, that is, natural disasters, shootings, killings, accidents, trials, etc.	N/A	1.3k TIMEX3 annotations; 7.3k Event annotations; 4 TLink types (Before, After, Contains, Overlap): 6.9k temporal relation annotations (TLinks)
Universal Decompositional Semantics Time (UDS-T) [29] (2019)	Categorical temporal relations are represented as real-valued relative timelines (on top of Universal Dependency parse trees)	Mapping of events to their likely durations and event pairs as real-valued relative timelines	Mixed Web documents (blog posts, newsgroup threads, emails, product reviews, and answers from QA websites) [38]	254.8k tokens [38]	32.3k Event annotations; 70.4k temporal relation annotations
TDG [39] (2020)	N/A (corpus)	Subset of TimeML + Temporal Dependency Graphs	500 WikiNews articles	Not reported	2.5k TIMEX3 annotations; 15.0k Event annotations; 4 TLink types (Before, After, Overlap, Includes): 28.4k temporal relation annotations (TLinks)
Maven-Ere [40] (2022)	N/A (corpus)	Subset of TimeML: TIMEX3, TLink	4480 Wikipedia articles	Not reported	25.8k TIMEX3 annotations; 6 TLink types (Before, Contains, Overlap, Begins_on, Ends_on, Simultaneous): 1216k temporal relation annotations (TLinks)
TimeBankNT [41] (2024)	Full timeline annotation (all possible TLinks) of the sparse TLink TimeBankDense [28] annotations	Subset of TimeML: TIMEX3, Event, TLink	36 news articles(subset of TimeBank 1.2)	13.3k (estimated)	0.3k TIMEX3 annotations; 1.7k Event annotations; 102.3k (104.8k) temporal relation annotations (TLinks)

^a^N/A: not applicable.

The early TimeBank corpus [25,26,28,29] witnessed the consolidation of TimeML over a decade. As an experimental vehicle, it is characterized by small document and token numbers as well as low amounts of annotated temporal data.

A simplified version of expressionally rich TimeML became the metadata backbone for TempEval [30-32,42,43], a series of time-focused shared task challenges run by NLP researchers within the framework of SemEval, a workshop series focusing on a broad range of semantic interpretation topics organized under the auspices of the Association for Computational Linguistics.

Shared tasks, such as TempEval or SemEval, have become a major driver of progress for NLP [44,45]. They are based on a common gold standard dataset on which several competing research group teams run their own systems against predefined tasks; their submitted solutions are, finally, compared against the gold standard, which yields a ranking of the teams involved based on their performance for specific tasks. Top-ranked teams are then given the opportunity at a closing workshop and invited to journal publications to feature the most successful methodologies—thus, continuously advancing the methodological repertoire of NLP on the basis of empirical evidence from benchmarking experiments.

The TempEval-3 corpus [31] was the largest in this series and contains 255 news articles, with roughly 95k tokens, selected from the TimeBank 1.2 corpus [25,26] (183/186 articles) and the Aquaint corpus (73 news reports) [33] (numbers slightly differ dependent on the cited literature); TimeBank-Dense [28,29], its relationally richer successor, comes with a total of 12,715 temporal relations (more than 2 times the number of the original TimeBank corpus). Interestingly, the second edition of TempEval [32] contained data not only for English, but also for Chinese, French, Italian, Korean, and Spanish, whereas the third edition [31] dealt with English and Spanish data only. A variant of TempEval was considered in the Timeline Task 4 of SemEval 2015, which featured cross-document analysis of news articles to construct timelines for events [34] described in a series of documents. For all 3 challenges in TempEval, 9 subtasks had to be tackled that fell into 3 categories: time expression identification (spans and TIMEX3 classes, such as Date, Time, Duration, etc), event expression identification (spans and Modality, Degree, Polarity, etc), and temporal relation identification (temporal relations, such as BEFORE, OVERLAP, AFTER, and a narrative container relation typed as CONTAINS).

In an intermediary phase, new corpora were generated featuring different text genres (eg, user-generated contents [35,36,46], Wikipedia articles [40]) and playing with different sets of temporal relations, yet with only insignificant growth of document sets or metadata volumes. One of the rare departures from strict adherence to TimeML can be found in the UDS-T corpus [46], where TimeML’s categorical temporal relations are represented as real-valued relative timelines (on top of Universal Dependency parse trees). UDS-T also stands for a trend towards increasing quantities of documents/tokens (>250k tokens) and metadata (>100k annotation items). Truly impressive breakthroughs in both of these dimensions have been achieved only recently by getting large numbers of crowdworkers involved, such as for Maven-Ere [40], which excels in, for example, more than a million temporal relation annotations. In addition, TimeBankNT [41] increases the number of metadata by an order of magnitude (>100k temporal relation annotations) relative to TimeBank-Dense. Almost all of the corpora in Table 1 incorporate newswire/newspaper documents only (with some exceptions mentioned above). In contradistinction, Table 2 features corpora with clinical contents.

**Table 2:** Time-focused (English only) clinical corpus resources.

Corpus/(challenge) task (Year)	Functional characterization of (challenge) task	Annotation language	Text genre	Size in tokens	Type/number of metadata
CLEF [47] (2009)	N/A^a^ (corpus)	TIMEX3 & self-defined set of 8 temporal relations (Before, After, Overlap, Is_included, Ended_by, Begun_ by)	50 clinical reports; 50 histopathology reports;50 imaging reports	Not reported	Clinical named entity types (Drug-or-Device, Intervention, Condition, Investigation, Locus, etc); 3.8k named entity annotations; clinical relation types (has_Target, has_Finding, has_Location, Modifies, etc); 2.4k clinical relation annotations; 0.5k temporal relation annotations (for 10 documents, only)
i2b2 2012 [48] (2013)	Spans and types/attributes of temporal expressions (TIMEX3), referring to Dates, Times, Durations (incl. Normalization to ISO^b^8601 standard); Spans and types/attributes of Events, including. Both clinical concepts, such as Problems, Tests, Treatments, and Events relevant to the patient’s clinical timeline, such as Admissions, Transfers between departments; Temporal relations between the clinical Events and temporal expressions (Before, After, Overlap)	Simplified version of TimeML adapted to clinical narratives and purposes (eg, the inclusion of clinical concepts, such as Problems, Tests, and Treatments, into Events) [49]	310 discharge summaries	About 178k tokens	2.4k (train) + 1.8k (test) annotation units for temporal expressions (TIMEX3); 15.6k (train) + 13.6k (test) annotation units for temporal Events; 33.5k (train) + 27.7k (test) annotation units for temporal relations; 94.6k annotation units, in total
THYME [50] (2014)	N/A (corpus)	ISO-TimeML (THYME- TimeML) [23]; TIMEX3, Event, 5 temporal relations (Before, Overlap, Begins_on, Ends_on, Contains)	1254 clinical notes and pathology reports (oncology: brain cancer, colon cancer)	Not reported	Subset of the THYME colon cancer corpus (107 documents): 1.4k annotation units for temporal expressions (TIMEX3); 15.8k annotation units for temporal Events; 7.9k annotation units for temporal relations; 25.1k annotation units, in total
Clinical TempEval-1, subbranch within TempEval as part of SemEval [51] (2015) and Clinical TempEval-2 [52] (2016)	Spans and types/attributes of temporal expressions (TIMEX3), referring to Dates, Times, Durations, etc; Spans and types/attributes of Events, including both clinical concepts, such as Problems, Tests, Treatments; temporal relations between the clinical events and the document creation time (Before, After, Overlap, Before-Overlap) and narrative container relations	Extension of TimeML for temporal expressions, medical events, and temporal relations between events and times (Styler et al [50] shows the details of extensions of TimeML)	591 clinical notes and pathology reports (colon cancer)	Not reported	7.9k annotation units for TIMEX3 time expressions; 78.9k annotation units for Events; 23.2k annotation units for temporal relations; 110.0k annotation units, in total
Clinical TempEval-3 [53] (2017)	Same tasks as in Clinical TempEval-1 and Clinical TempEval-2	Same annotation language as in Clinical TempEval-1 and Clinical TempEval-2	Same colon cancer dataset as in Clinical TempEval-1 and Clinical TempEval-2 + a second dataset (595 documents) with focus on brain cancer	Not reported	(Virtually the same number of annotation items for colon cancer as in Clinical TempEval-1 and Clinical TempEval-2) 6.6k annotation units for TIMEX3 time expressions; 48.9k annotation units for Events; 7.3k annotation units temporal relations; 62.8k annotation units for colon cancer, in total; 172.8k annotation units; in total.
n2c2 [54], follow-up event of i2b2 2012 (2023)	Identification of medication change (Event): determine the Action, Negation, Temporality, Certainty, and Actor for any change events (Context)	Categories for temporality (Past, Present, Future, Unknown) are considerably coarser in granularity than those from the i2b2 2012 Shared Task and are not grounded in TimeML	500 clinical notes (discharge summaries, correspondence notes)	About 308k tokens (estimated from the study by Kumar et al [55])	About 1.7k annotation units
CALEX[56] (2019)	N/A (corpus)	TimeML-based TIMEX3 (Date, Time, Duration, Frequency) plus an extension (CALEX) for timeline construction based on calendar expressions	180 discharge summaries, 53 psychiatry reports, 70 pediatrics reports, 75 emergency room reports, 378 clinical reports, in total	55,433 (discharge summaries); 67,569 (psychiatry); 36,675 (pediatrics); 52,041 (emergency), 211.7k clinical reports, in total	2378 TIMEX3 annotations

^a^N/A: not applicable.

^b^ISO: International Organization for Standardization.

The CLEF corpus [47] was the first clinical time-focused dataset ever, with limited size though (150 documents, <7k annotated items from which only a small fraction cover temporal information), restricted to TIMEX expressions, yet with a rich repertoire of named entities, (self-defined) semantic and temporal relations. It became the forerunner of the seminal THYME corpus [50], a flagship enterprise that, unlike CLEF, fully adhered to TimeML representation conventions and came with roughly 1.3k clinical reports and slightly more than 25k temporal annotations on a 107-document subset thereof.

Fortunately, the idea of challenge competitions also attracted clinical NLP researchers. Inspired by TempEval, temporal aspects were targeted within the i2b2 2012 Shared Task on Temporal Relations in Clinical Text [48], here with focus on time information contained in clinical reports. Participating systems were required to extract virtually the same types of temporal knowledge as those targeted by TempEval:

1. Spans and types/attributes of temporal expressions, referring to Dates, Times, Durations, or frequency phrases in the clinical text (subsequently, the values of the extracted TIMEX temporal expressions had to be normalized to the ISO8601 specification standard).
2. Spans and types/attributes of clinically significant events, including both clinical concepts (ie, named entities) such as Problems, Tests, Treatments, and ClinicalDepartments, and events relevant to the patient’s clinical timeline, such as Admissions, Transfers between departments.
3. Temporal relations between the clinical events and temporal expressions.

For these challenge tasks, the i2b2 2012 Shared Task organizers added a temporal annotation layer to the dataset, 310 discharge summaries (about 178k tokens), that had already been used in previous i2b2 rounds. For annotation purposes, a simplified version of TimeML was taken as a starting point, but had to be adapted to better suit clinical narratives and purposes (eg, the inclusion of clinical concepts, such as Problems, Tests, and Treatments, into Events) [49]. The i2b2 2012 Shared Task corpus, finally, is accessible on the basis of signing a data use agreement and comes (for training) with 2366 annotation units for clinically relevant temporal expressions (TIMEX3 data), 15,567 annotation units for clinically relevant temporal events, and 33,543 annotation units for clinically relevant temporal relations; train and test data sum up to almost 95k annotation units. This landmark corpus exceeded previous annotation efforts in this field (most notably CLEF [47]) both in terms of the number of annotated documents and annotation units by a large margin and became a benchmark for future research.

Recently, a time-wise much narrower shared task devoted to medication change events documented in clinical notes was run within the context of n2c2, the follow-up event of i2b2 [54]. Participants had to identify medication change (Event) and determine the Action, Negation, Temporality, Certainty, and Actor for any change events (Context). The categories for temporality (Past, Present, Future, Unknown; about 1.7k annotation units) are considerably coarser in granularity than those from the previous i2b2 2012 Shared Task and are not grounded in TimeML. An annotation effort that extends TIMEX3 expressions for timeline construction based on calendar expressions is reported by Viani [56].

Complementary to these activities, within the TempEval branch of SemEval, a clinical TempEval subbranch was established. In the first 2 runs of this medically focused TempEval challenge [51-53], roughly 600 deidentified clinical notes and pathology reports from colon cancer patients at Mayo Clinic were manually annotated with an extension of TimeML for temporal expressions, medical events, and temporal relations between events and times (details of the annotation process and extensions of TimeML as part of the THYME corpus are described by Styler et al [50]). In the third run of TempEval [53], this dataset was complemented by a second one with a focus on brain cancer, as the organizers wanted to explore the potential for domain adaptation, that is, how do systems trained on colon cancer data perform when tested on brain cancer data?

The TimeML-annotated corpus from TempEval is available on a data use agreement basis as well, and comes (for colon cancer) with roughly 83k annotation units in its training (50%), and development (25%) splits, hence contains around 110k annotation units together with the held-out test set (25% of the entire dataset). For brain cancer, 48k annotation units are available for training (50%) and development (25%), and 15k in the test split, summing up to 63k, in total. Thus, the corpus from TempEval-3 exceeds the i2b2 2012 Shared Task corpus by slightly less than a factor of 2 in terms of annotation units and figures as the second milestone for temporal clinical corpora for the English language.

All of the above-mentioned corpus building activities almost only cover the English (clinical) language. Since our work deals with German language data, Table 3 gives an overview of the few efforts to deal with nonclinical German from a temporal perspective.

**Table 3:** German time-focused nonclinical resources (Corpora and Taggers).

Resource type	Name of resource (Year)	Functional characterization	Annotation language	Text genre	Size in tokens	Type/number of metadata
Corpus	EuroParl [57] (2008)	Time annotation mappings in parallel corpora: English → German	Subset of TimeML: TIMEX3, Events, no temporal relations	Transcribed speeches in the European Parliament	960k bisentences (train) and 0.3k bisentences (dev/ test)	Not reported
Corpus	WikiWarsDE [58] (2011)	N/A^a^	TIMEX2 [22], a subset of TimeML restricted to dates, time expressions, and durations)	22 German Wikipedia articles about famous wars in history corresponding to the English selection in WikiWars [59]	95.6k tokens	2.2k TIMEX2 annotation units
Corpus	KRAUTS [60] (2018)	N/A	TIMEX3	192 newspaper articles	75.7k tokens	1.1k TIMEX3 annotation units
Time tagger	HeidelTime [61] (2013)	Rule-based tagger using gazetteers and regular expressions	TIMEX2, TIMEX3 subset of TimeML plus normalizations into ISO^b^ 8601 standard	22 German Wikipedia articles about famous wars in history corresponding to the English selection in WikiWars [59]	100.7k tokens	2.2k TIMEX2 annotation units
Time tagger	HeidelTimeext [62] (2022)	Extended rule-based tagger using gazetteers and regular expressions	TIMEX3	Mix of 10 parliament protocols, 10 books, 766 newspaper and 10 scholarly articles, 10k Wikipedia sentences	5.121k tokens	56.8k/53.8k TIMEX3 annotation units (automatically generated)

^a^N/A: not applicable.

^b^ISO: International Organization for Standardization.

Three German-language corpora have been built with a focus on time information. EuroParl [57] collects transcribed speeches (roughly 1m bisentences) from the European Parliament. TimeML-conformant temporal annotations are automatically mapped from English source texts to German targets. WikiWarsDE [58] assembles 22 Wikipedia articles on famous wars; its time representations are restricted to TIMEX expressions. So is the annotation in KRAUTS [60] based on 192 newspaper articles. For all 3 corpora, token counts do not exceed 100k, and at most, roughly 2k manual gold standard annotations are provided (HeidelTimeext [62] offers a substantially larger yet automatically generated silver standard). Overall, these are, in comparison with the numbers in Table 1, tiny corpora with low annotation density and limited representational expressiveness (with the exception of Spreyer and Frank’s study [57]).

Two versions of the same time tagger also deserve mention. HeidelTime [61] was originally built on English data and then augmented for tagging German data. HeidelTime is a classical rule-based system using regular expressions and additional gazetteers. The step from HeidelTime to HeidelTimeext [62] comes with an augmented and updated rule set, an increase of the document size and token number (more than 5m tokens), and the number of temporal annotations (more than 50k items). However, in both cases, the representational expressiveness is limited to TIMEX expressions.

The state of German-language clinical corpora with temporal metadata is basically a short story summarized in Table 4. The MACCS corpus [63] is made of slightly more than 1.7k discharge summaries and clinical notes from the nephrology domain whose temporal annotations are based on a corpus-specific, that is, non–TimeML-conformant annotation language. Our work, instead, is fully TimeML-compliant and features 2 different corpora. 3000PA_J_ comes with 1100 real clinical documents, 1.7M tokens, and 120k temporal annotations, whereas GraSCCo is a much smaller corpus product featuring 63 synthetic documents, 44k tokens, and almost 12k temporal annotation units. Due to rigid data privacy legislation in Germany, 3000PA_J_ cannot be made publicly available, whereas GraSCCo can (it consists of fictitious clinical data). With roughly 131k annotation items, however, both corpora not only exceed all German-language corpora (whether clinical or nonclinical) in terms of temporal metadata but also all clinical English time-focused corpora, with one exception—only the million-scale nonclinical Maven-Ere corpus [40] offers a (much) larger number of temporal annotations.

How do 3000PA_J_ and GraSCCo compare with other non-English clinical corpora annotated with temporal metadata? Table 5 lists such textual resources whose size exceeds 50 documents and 1k annotation units. Perhaps, the closest in kinship is the French MERLOT corpus [64] that also adheres to TimeML specifications but falls behind by orders of magnitude both in terms of tokens and the amount of temporal metadata. Three other French corpora [65-67] are much smaller than MERLOT and also contain fewer temporal annotations; only Bannour et al [66] adheres to TimeML, including temporal relations. From 4 Chinese corpora, only the work of Liu et al [68] contains TimeML specifications for TIMEX3 expressions and temporal relations, yet on a much smaller scale than 3000PA_J_, both document, token, and metadata-wise. Pan et al [69] and Liu et al [70] focus on TIMEX3 expressions only (Liu et al [70] offering the largest corpus in terms of the number of documents, yet not of the number of annotation items), whereas Hu et al [71] use a self-supplied time ontology for annotation. Swedish [72], Portuguese [73], Spanish [17], and Italian [74] resources are tiny in terms of the number of documents and annotation items they contain (in the range of less than some hundred documents) and the number of temporal relations (less than 5k, if at all). Finally, Cheng et al [75] contain 2.6k THYME-conformant temporal relation annotations from a Japanese corpus based on slightly more than 1k documents. Another Japanese corpus, MedTxt-CR-JA [76], comes with 27.4k temporal relation annotations that are inferred from 2.6k explicitly annotated start-point annotations. TIMEX3-only annotations are provided by Hamon et al [65], Jeyafreeda et al [67], Pan et al [69], and Velupillai [72]. With the exception of the multilingual E3C corpus [77] (composed of French, Spanish, Basque, and Italian clinical case reports which differ significantly in style and format from real clinical reports), none of these corpora is publicly available.

**Table 4:** German time-focused clinical corpus resources with our own contribution highlighted.

Resource type	Name of resource (Year)	Annotation language	Text genre	Size in tokens	Type/number of metadata
Corpus	MACSS [63] (2016)	Corpus-specific, that is, non-TimeML-conformant temporal annotations: time points, dates, temporal courses	118 discharge summaries;1607 clinical notes from the nephrology domain (kidney transplantations)	89.7k tokens; 68.5k tokens	Not reported
Corpus	3000PA_J_ (our work)^a^	TimeML:TIMEX3, Events, Six temporal relations (Before/After, Contains, Overlap, Begins_on, Ends_on)	1100 real clinical documents, mostly discharge summaries	1.7M tokens^a^	13.2k annotation units for TIMEX3 time expressions; 46.9k annotation units for Events; 59.1k annotation units for temporal relations; 119.2k^a^ annotation units, in total
Corpus	GraSCCo (our work)^a^	TimeML:TIMEX3, Events, Six temporal relations (Before/After, Contains, Overlap, Begins_on, Ends_on)	63 synthetic clinical documents	44k tokens^a^	1.2k annotation units for TIMEX3 time expressions; 4.5k annotation units for Events; 5.8k annotation units for temporal relations; 11.6k^a^ annotation units, in total

^a^Our own contribution.

**Table 5:** Non-English and non-German time-focused clinical corpus resources.

Natural language (ISO^a^ 639 codes)	Name of resource (Year)	Annotation language	Text genre	Size in tokens	Type/number of metadata
FR	No name [65] (2014)	TIMEX3 expressions (plus normalizations)	182 clinical records (train) and 120 records (test, 25 with reference annotations), 302 records, in total	Not reported	Not reported
SV	Stockholm EPR [72] (2014)	TIMEX3 expressions	112 clinical notes from an ICU^b^ unit	≈50k (estimate)	<2k TIMEX annotations
ZH	CMedTEX [70] (2016)	Simplified TimeML TIMEX3 expressions: Date, Time, Duration, and Frequency (plus ISO8601 normalizations)	1778 clinical notes	Not reported	46.6k TIMEX3 annotations
PT	No name [73] (2018)	TimeML TIMEX3: Date, Time, Duration, Set (plus ISO8601 normalizations)	130 discharge summaries	Not reported	Not reported
FR	MERLOT [64] (2018)	Simplified TimeML: Date, Time, Duration, and Frequency; 6 TimeML-compliant temporal relations: Before, Begins_on, Ends_on, During, Overlap, Simultaneous	500 discharge summaries, procedure reports (eg, radiology reports), physician letters, and prescriptions	148.5k	3.9k temporal entity annotations; 4.6k temporal relation annotations
ZH	No name [68] (2019)	Three TimeML-compatible temporal relations: Before, After, Simultaneous	563 clinical notes	Not reported	4.0k temporal expressions (ako TIMEX3 expression); 4.0k time intervals (relations)
IT	No name [74] (2019)	THYME-conformant clinical events (Problems, Tests, Treatments, Occurrences) and temporal relations (Before, Before/Overlap, Overlap, After)	75 clinical reports	57,263	4.4k temporal relation annotations
ES	No name [17] (2020)	TIMEX3 expressions (plus expression normalization) and TimeML-noncompliant temporal relations	50 clinical notes and 50 clinical reports for TIMEX3 identification & 100 clinical notes and 100 clinical reports for temporal relation identification, 300 documents, in total	Not reported	TIMEX3 annotations not reported; 200 temporal relations
ZH	TNorm [69] (2020)	TimeML-compliant TIMEX3 (plus expression normalization) only, no temporal relations	900 discharge summaries	Not reported	12.1k TIMEX3 annotations
ZH	No name [71] (2022)	Self-designed Clinical Time Ontology as a basis for an OWL-based temporal logic, with 11 temporal classes (various types of Instants, Intervals, Instant and Interval Collections, and Modifiers) and 16 temporal relations (extension of Allen’s 13 relations set)	300 case reports containing 3000 clinical statements (presumably sentences)	Not reported	3.5k temporal entity annotations; 3.0k temporal relation annotations; 6.5k annotation units, in total
JA	No name [75] (2022)	THYME-compliant, simplified annotation schema: TIMEX3 + 5 temporal relations: On, Before, After, Start, Finish (complemented by 5 medical relations: Change, Compare, Feature, Region, Value)	1000 radiography interpretation reports of lung cancer + 156 medical history reports of idiopathic pulmonary fibrosis	Not reported	2.6k temporal relation annotations
FR, ES, IT, EU	E3C [77] (2023)	THYME-conformant annotation: TIMEX3 expressions, Events, temporal relations	81 French and Spanish, 86 Italian, and 90 Basque clinical case reports + 615 clinical case reports with silver standard (automatic) annotations	French: 25,196; Spanish: 24,681; Italian: 24,319; Basque: 22,505 + >200k (silver standard)	French: 0.3k TIMEX3, 4.3k Event, 4.0k temporal relation annotations; Spanish: 0.4k TIMEX3, 4.8k Event, 4.7k temporal relation annotations; Italian: 0.3k TIMEX3, 3.4k Event, 1.2k temporal relation annotations; Basque: 0.6k TIMEX3, 7.9k Event, 8.0k temporal relation annotations
FR	No name [66] (2023)	Subset of TimeML Document; Creation Time and 5 temporal relations: Before, Before_Overlap, Overlap, and After + TemporalReference (noncompliant with TimeML)	220 (train) + 57 (test) clinical reports	Not reported	2.9k temporal relation annotations
JA	MedTxt-CR-JA [76] (2024)	Start-point ordering of events from which 3 temporal relations can be inferred: Before, Equal, and After	62 (from 148) case reports	Not reported	27.4k temporal relation annotations (based on 2.6k explicitly annotated start-point annotations)
FR	No name [67] (2024)	Corpus-specific, that is, non-TimeML-conformant temporal annotations: Date, Time, Frequency, Duration, and Age	150 (train) + 50 (test) clinical notes	Not reported	0.4k temporal entity annotations

^a^ISO: International Organization for Standardization.

^b^ICU: intensive care unit.

### Goals of This Work

As stated above, temporal knowledge, its annotation, and automatic extraction in the medical domain have almost only been researched in depth in the Anglo-American language community. The non-English clinical language community is basically a low-/nonresource area that, with only a few exceptions, generally lacks temporal metadata. In this article, we propose a 2-fold solution, specifically responding to the strict privacy legislation constraints in the European part of the world.

The unique contributions of this article are the following: We provide temporal annotations for German clinical documents, including quality checks via interannotator agreement (IAA) metrics. More specifically, we supply temporal annotations for a nondistributable corpus composed of real clinical documents and a publicly distributable corpus composed of synthetic clinical documents. Furthermore, we train transformer-based baseline taggers to automatically extract temporal information encoded in German clinical reports and notes.

## Methods

### Overview

**Figure 2:**
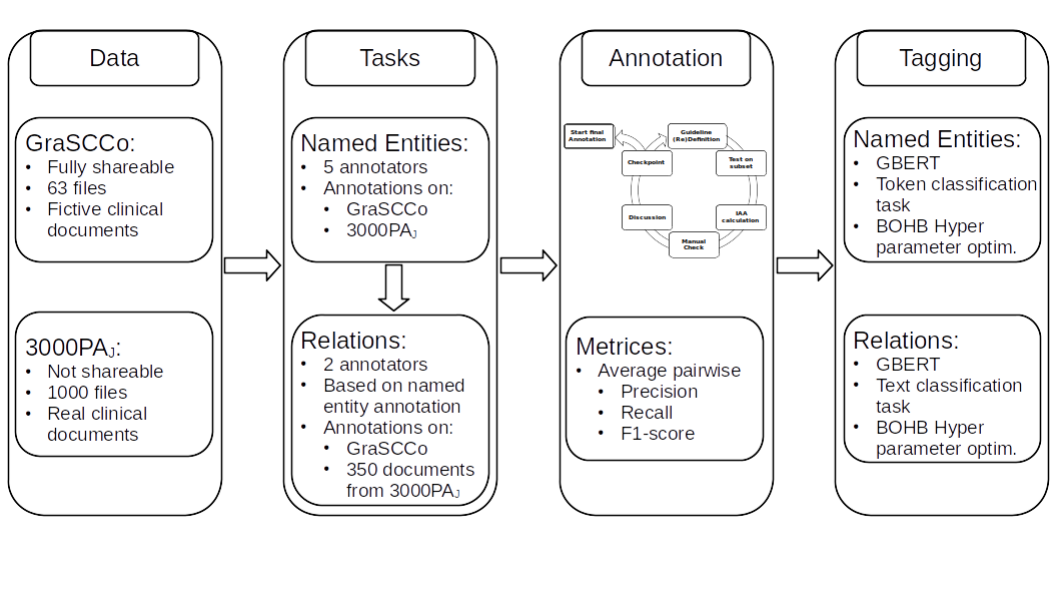
Overview of the experimental setup and workflow. BOHB: Bayesian Optimization and Hyperband; GBERT: German BERT model.

In this section, we will give a detailed overview of our 2 clinical corpora, the annotation schema for temporal named entities and relations, the annotation process and its evaluation, as well as the presentation of our baseline time tagging model for clinical documents. Figure 2 summarizes our experimental setting and illustrates the key steps in our workflow.

### Ethical Considerations

The usage of 3000PA_J_ is based on the approval by Jena University Hospital ethics committee (4639-12/15) and its data protection officer. The data was collected during the routine clinical care of adults at the intensive care unit or internal medicine ward. All persons were deceased at the time of data collection, thus no informed consent was needed for local data analysis according to the German law at that time. All data were hosted, annotated, and processed at the premises on secure servers with access restrictions and regular data protection training of the annotators. For GraSCCo, no ethics approval is needed as this dataset contains only fictional data written in the style of real clinical documents.

### Clinical Data Resources

For temporal annotation, we used the 2 corpora already introduced in Table 4, namely 3000PA_J_ and GraSCCo. 3000PA_J_, a subset of the 3000PA corpus [78], consists of about 1100 real, that is, authentic, clinical documents, mostly discharge summaries, with roughly 145k sentences and 1.7m tokens hosted by the Jena University Hospital under strict access restrictions. In effect, this means that according to German data protection legislation, this dataset cannot be made available for public use outside the walls of the Jena University Hospital. 3000PA_J_ has already been annotated at multiple semantic layers, including section headings [79], Personally Identifiable Information [80], medications [78], and major nontemporal clinical named entities, namely diagnoses, findings, and symptoms [81].

As an alternative to siloing much-wanted clinical data in the barred 3000PA_J_ dataset, we developed GraSCCo [82], a synthetic twin corpus which is open for use to the entire clinical NLP community without any access restrictions. Albeit being a tiny document collection with only 63 German multiply-alienated and noise-infused real documents with about 5.4k sentences and 44k tokens, it is available via the Creative Commons license on Zenodo [83]. As a noteworthy remark, the authors have already gathered preliminary evidence that GraSCCo may act as a reasonable substitute for 3000PA in terms of clinical language use and genre patterns [82]. This observation is fully consistent with other experimental results for various languages, which show that synthetic corpora can indeed replace real clinical corpora with only marginal quality loss regardless of the application tasks and clinical text genres chosen (see, eg, the evidence found for English [84-86], French [87], and Norwegian [88]).

### Temporal Annotation

#### Overview

**Figure 3:**
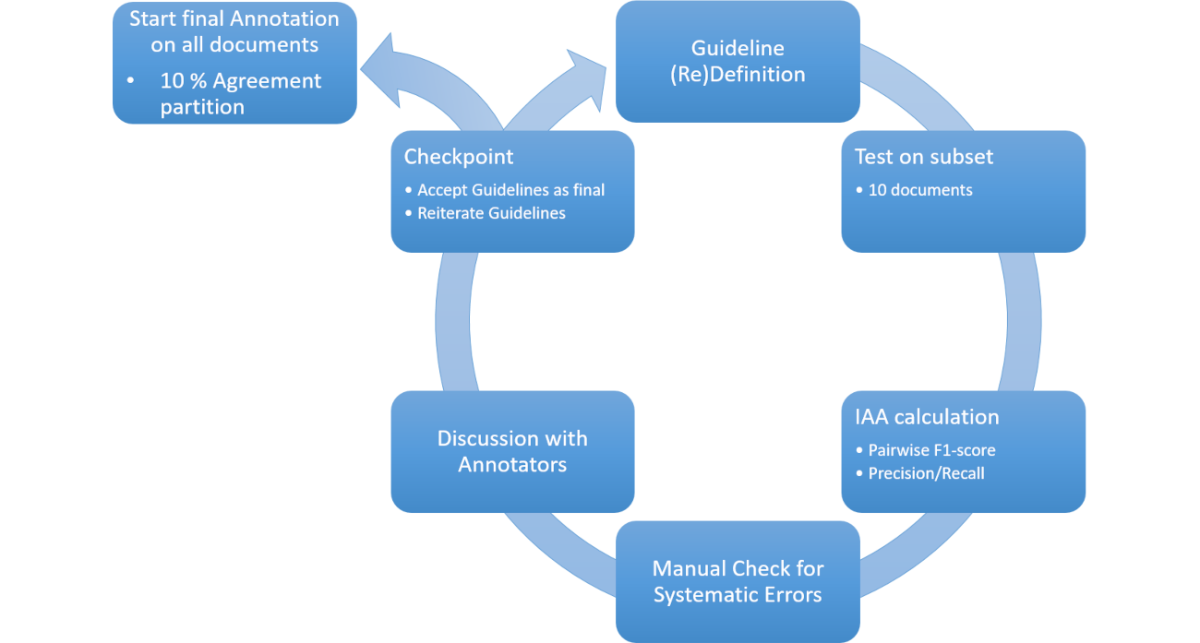
Schema of the iterative workflow for annotation guideline development. The optimization cycle is repeated until a stopping criterion is reached on a document subset, mostly a predefined IAA threshold or the number of iterations. The final guideline is subsequently used to annotate the entire corpus in the production phase. IAA: interannotator agreement.

Manually generating temporal annotations is well-known as a notoriously complex and time-consuming task [49,50]. Hence, to avoid cognitive overload of the human annotators, we split our annotation campaign into 2 separate phases—an entity annotation and a relation annotation phase. Both followed the same schema (Figure 3 depicts one phase equals a complete cycle) and were performed by at least 3 different annotators. This organizational scheme follows the widely acknowledged annotation conventions established by James Pustejovsky and Amber Stubbs [89]. Generally speaking, at least 3 independent annotators are necessary to run a proper statistical analysis. All of our annotators were qualified medical students who had already passed their first medical exam after 2 years of study (Physikum, in German).

Each annotation cycle was divided into a training-only and a production annotation phase. During the training phase, all annotators received the same partition of the entire dataset for annotation, usually about ten documents. Once this batch was completed, their annotations were checked both automatically via an IAA metric based on the average of the pairwise *F*_1_-scores, and manually to identify and resolve individual/systematic errors and misunderstandings. The results of the latter phase were then discussed within the annotation team and led to changes in the annotation guidelines, if needed (typical shortcomings arising from premature annotation guidelines are a lack of clarity or ambiguity of instructions, instruction gaps, etc). After that, a new subset was drawn and, again, annotated by all annotators. These training iterations continued until the *F*_1_-score, primarily the pairwise *F*_1_-score as well as the averaged *F*_1_-score, stabilized and reached a reasonable, that is, community-wide accepted, plateau (in the range of 0.9 on a scale of 0 to 1.0 agreement) [90]. After this preparatory phase, the final guidelines were frozen and applied to the entire dataset, again, with a randomly chosen partition as the agreement portion, usually 5% to 10% of all documents. These documents will be called agreement documents, as they are the documents on which the IAA is calculated. The final *F*_1_-score was determined on these agreement documents and reported as the final agreement value.

Once the entity annotation task was completed, we unified all annotations of all agreement documents. Thus, label disagreements and missing annotations were detected. These merged entity annotations with all their identified disagreements were then used for the second phase, the relation annotation, and manually corrected by the human annotators. The annotators were also encouraged to modify/correct entity annotations if they disagreed with the results of the first entity-focused round.

#### Temporal Entity Definition

Our definitions of time-related entities are identical to THYME [50]. Accordingly, those entities are defined as follows:

- EVENT: Any token or phrase that is related to things that occurred or happened.
- TIMEX3: Any token or short phrase that denotes a time expression of the following subtypes:
  Date: Expressions or phrases that describe a time with accuracy up to a day (06/2020, next Wednesday),
  Time: A specific time of the day (12 am, midnight),
  Duration: A time frame (until yesterday, for 10 minutes),
  Set: A recurring event (twice a day, 1-0-0),
  Quantifier: The number of times something has happened or occurred, needs to be paired with an EVENT (second cycle of chemotherapy),
  PrePost: Expressions like postoperative or premenarche.

**Figure 4:**
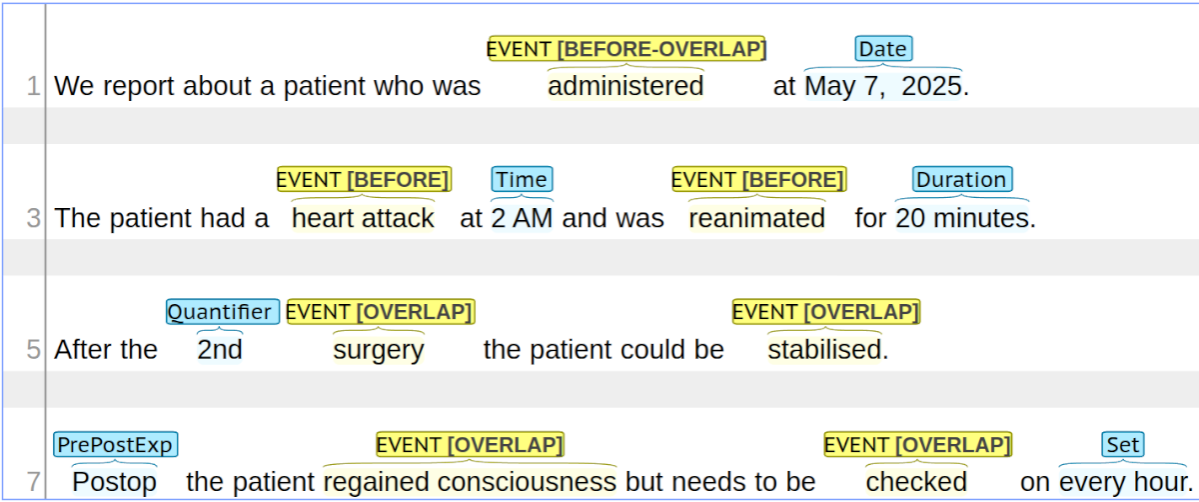
Sentences to illustrate the annotation of entities annotated with the BRAT annotation tool. Yellow indicates EVENTS, blue stands for TIMEX3 temporal expressions. The labels are written over the marked words or phrases.

Additionally, entities annotated as an EVENT needed to be marked with one of the following tags according to their point in time with respect to the reported hospital stay: BEFORE (if something happened prior to admission), BEFORE-OVERLAP (if something occurred before but is still present at the time the patient was admitted to the hospital), OVERLAP (if something happened during the hospital stay) and AFTER (if occurrences will happen in the future after the discharge). An example of said annotated entities can be found in Figure 4.

#### Temporal Relation Definition

Temporal entities themselves do not contain sufficient information for the adequate construction of timelines. Without context or ordering information, most of the time-relevant information is missing. Accordingly, temporal relations between those entities have to be determined. All of the relation types we chose are a subset of Allen's interval algebra [21]. We followed the definitions in THYME [50] and used the following relation types:

- BEFORE (X,Y) X happened entirely before Y
- (AFTER (Y,X) is treated as the inverse of BEFORE (X,Y))
- CONTAINS (X,Y) Y happened entirely during X
- OVERLAP (X,Y) X and Y share a common partition of time
- BEGINS-ON (X,Y) X started with Y
- ENDS-ON (X,Y) X finished with Y

**Figure 5:**
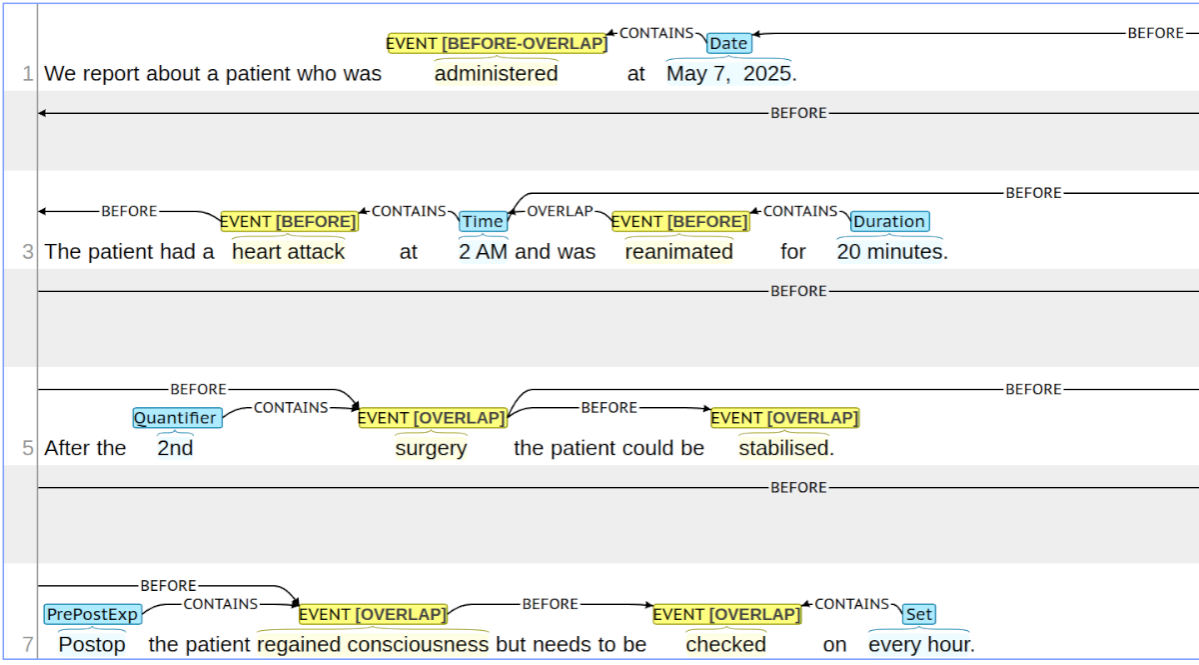
Extension of the already annotated named entities from Figure 4 with temporal relations using the BRAT annotation tool. The arrows indicate the relation together with its relation type.

In contrast to THYME and more in line with the i2b2 2012 guidelines, we have instructed our annotators to link each entity to at least one other entity in such a way that each entity is connected to every other entity via a path. This way, we ended up with a connected temporal graph where the entities are the vertices and the relations are represented as edges. This condition is crucial, however, to create a timeline of the entire hospital stay in later processing stages. In contrast to i2b2 2012, we did not use direct connections with the document time or hospital stay (time). This was already covered in the named entity phase by encoding the respective Event tag. Figure 5 demonstrates the application of our annotation conventions to the sentences with already annotated named entities from Figure 4.

#### Evaluation Methods

To determine the IAA scores in our temporal named entity annotation campaign, we calculated the mean of the pairwise precision, recall, and *F*_1_-score for the annotated entities. The implementation we used can be found on GitHub [91].

In contrast to temporal named entities, temporal relations need a different treatment for their evaluation. Every entity has temporal connections to every other entity. However, annotating the entire fully connected temporal graph is not feasible, since the number of temporal relations grows quadratically with the number of event mentions (by anchoring events in time, however, annotation of temporal relations indeed scales linearly with the number of events [92]). Thus, each annotator was encouraged to annotate the least possible number of relational edges, resulting in something akin to a minimum spanning tree. To guide the annotators towards this goal, they were instructed not only to connect named entities but to think in terms of units of meaning. These units consist of smaller groups of events, such as the timeline of a surgery, the family history, or the events that happened before admission. These smaller units should then be linked to each other, creating a larger group. This should be repeated until all larger units of meaning are linked into one large, comprehensive timeline. If possible, the anchors for these temporal units should be TIMEX3 entities, preferably Dates. A fictitious example for these linkage conventions can be found in Multimedia Appendix 1.

These conventions result in a number of different graphs that, in the worst case, might share no edges at all. This would lead to a much worse agreement score than justified. The solution we propose here is to use inference and temporal closure [93]. We infer the missing edges for each annotator and are thus able to calculate a pairwise agreement score (namely precision, recall, and *F*_1_-score; see below). Based on the overlap of temporal relation definitions, we used a slightly adjusted version of the THYME evaluation script. The changes included adjustments to our annotation format (BRAT) [94], extending the evaluation, and the visualization of annotations.

#### Training and Evaluation of a Baseline Temporal Tagger

To demonstrate the usefulness of the outcome of our annotation campaign and to determine the first baseline for the automatic temporal tagging of German clinical documents, we used state-of-the-art transformer-based classification technology, that is, BERT (Bidirectional Encoder Representations from Transformers)-ish language models. For German, the most recent and best-performing language model is the German BERT model (GBERT) [95]. For our experiments, we used the base model for each of the downstream tasks we specified. To automatically optimize hyperparameters for each task, we used Bayesian Optimization and Hyperband [96] (Multimedia Appendix 2). Our experiments incorporate the huggingface library [97], as well as the Bayesian Optimization and Hyperband implementation of the Microsoft NNI framework [98]. This configuration was applied to and evaluated on the nonsharable 3000PA (ie, 3000PA_J_) and the publicly sharable GraSCCo corpus. The ground truth data used for training, testing, and evaluation were taken from the annotated documents without further correction. If the document was part of the agreement partition, and thus had been annotated by all annotators independently, we randomly selected one annotation variant from one annotator.

#### Evaluation Metrics

For the statistical evaluation, we apply the following metrics.

Precision (P), also known as positive predictive value, is the ratio between the gold-standard matching annotations of all named entities or temporal relations for each label (true positives) and all annotated named entities or temporal relations for each label (true positives + false positives).



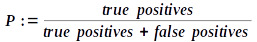



Recall (R), also called sensitivity, is the ratio between the gold-standard matching annotations of all named entities or temporal relations for each label (true positives) and all annotated named entities or temporal relations for each label (true positives and false negatives).



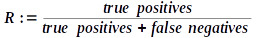



*F*_1_-score is defined as the harmonic mean of precision and recall.



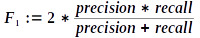



Accuracy (Acc) is defined as the ratio between the gold-standard matching annotations (true positives and true negatives) and all annotations, regardless of their annotations being true or false (sum of annotated entities).



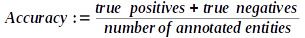



All of these measurements were calculated pairwise between all possible annotator pairs. The final agreement score for the labels or the entire corpus reports the averaged pairwise agreement scores.

## Results

### Overview

We present the results of our experiments, distinguishing between manual temporal named entity and temporal relation annotations, as well as the evaluation of the GBERT baseline tagging model that automatically generates temporal tags for clinical documents.

### Annotation of Temporal Named Entities

In the first cycle of our 2 annotation campaigns, we annotated temporal named entities on both 3000PA_J_ and GraSCCo. The results presented here were calculated on the corrected named entity annotations after the relation annotation campaign with its named entity correction subtask.

As can be seen in Table 6, we achieved pairwise *F*_1_-scores above 0.8 for nearly all named entity types. Moreover, our overall agreement even exceeds an average *F*_1_-score above 0.9. When looking at the token-level evaluation (which is less strict), we achieved an overall agreement average *F*_1_-score for 3000PA_J_ of 0.912 and 0.910 for GraSCCo.

**Figure 6:**
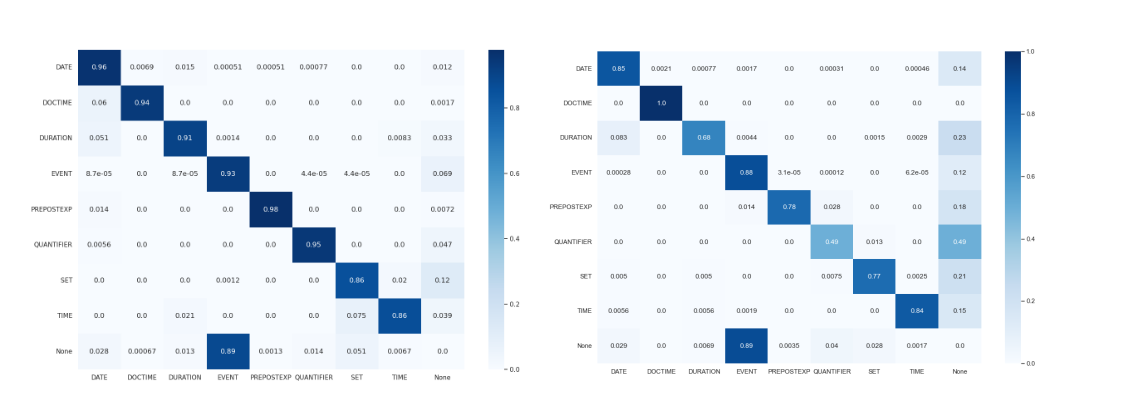
Confusion matrices for temporal named entity types after correction, with 3000PAJ (left) and GraSCCo (right). The color and intensity indicate the relative number of entity pairs (with 1.0 being all and 0.0 no existing pairs). Agreeing pairs (both annotators chose the same label) can be found on the main diagonal.

On both datasets, the entity type Duration yielded slightly worse scores. There is only one major outlier in terms of IAA in the 3000PA_J_ results, namely Quantifier, both in terms of *F*_1_-score and SD. Interestingly, this is not observed in GraSCCo. When looking at the confusion matrix of the named entities of both datasets in Figure 6, we are able to explain this deviant behavior.

As can be seen in the confusion matrices for entity types of both corpora in Figure 6, the main reason for mismatching labels was mostly missing annotations, labeled “None” in the confusion matrix. The Event type, in particular, was often only annotated by one annotator. This behavior can be observed for both corpora. Interestingly, in 3000PA_J_, the type Quantifier was also often missed by one of the annotators. The most often confused label pair was Date and Duration. Some translated examples for confused label pairs are:

- {since 2017}DURATION vs since {2017}DATE
- {the entire night}DURATION vs the entire {night}DATE
- {after about 30 minutes}DURATION vs after about {30 minutes}TIME
- {day of self-harm}DATE vs day of {self-harm}EVENT

Some systematic errors also appear in the distinction between Duration and Date. Most of the mismatches were grounded in some form of trigger word, such as “since” or “after”. However, it needs to be stressed that these kinds of errors did not occur too often relative to all cases, as can be seen in the confusion matrices in Figure 6. Mismatches between labels other than the ones described above are very rare (per milli range).

**Table 6:** Absolute number and relative number in percentage of named entities, mean pairwise F1-score with SD for each entity type of the temporal named entity annotation campaign for 3000PAJ and GraSCCo, respectively, as well as the overall agreement for each dataset, which also includes the nonannotated tokens.

Named entity	3000PA_J_				GraSCCo			
	Absolute number	Percentage (%)	*F*_1_-score	SD	Absolute number	Percentage (%)	*F*_1_-score	SD
Date	9099	15.13	0.903	0.055	663	11.51	0.947	0.020
DocTime	886	1.47	0.988	0.007	92	1.60	0.944	0.031
Time	593	0.99	0.893	0.058	3	0.75	0.852	0.075
Duration	824	1.38	0.776	0.083	138	2.40	0.747	0.091
Set	1107	1.84	0.822	0.081	158	2.74	0.802	0.107
Quantifier	563	0.94	0.550	0.209	118	2.05	0.938	0.023
PrePost	146	0.24	0.831	0.041	24	0.42	0.932	0.041
EVENT	46,909	78.02	0.910	0.017	4523	78.54	0.906	0.053
Total	60,127	100.0	0.834	0.068	5759	100.0	0.883	0.055
Overall Agreement	—^a^	—	0.904	0.024	—	—	0.906	0.047

^a^Not applicable.

### Annotation of Temporal Relations

Because of their complexity and the human efforts required for this task, temporal relations were annotated only on a randomly chosen subset of 350 documents of the original 3000PA_J_ dataset, whereas all 63 documents of the GraSCCo dataset were dealt with. Both datasets were annotated by the same 4 annotators. The resulting IAA scores incorporating the aforementioned inference rules are depicted in Table 7. Because of the nature of points in time and their relation to each other (they form a fully connected temporal graph), relations between 2 named entity pairs always have a relation type, even though it might be automatically inferred rather than manually annotated.

As can be seen in the total count of relation types, in GraSCCo all entities seem to be connected with each other, as the number of relation types is slightly larger (40 relations more than necessary) than the total number of entity types. But in 3000PA_J_ we seem to miss a total of about 1k relations, which means approximately 3 relations per document. However, given the total number of about 60k annotated relations, this is just 1.76% of the anticipated number of relations.

**Figure 7:**
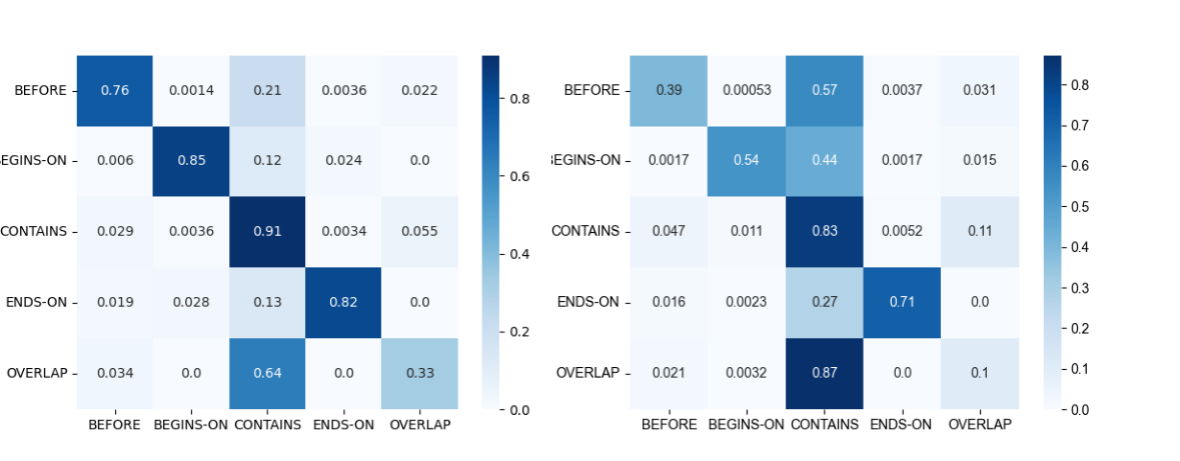
Confusion matrices for temporal relation types, with 3000PAJ (left) and GraSCCo (right). The color and intensity indicate the relative number of relation label pairs (with 1.0 being all and 0.0 no existing pairs). Agreeing pairs can be found on the main diagonal.

The confusion matrices for the 5 temporal relation types of both datasets are depicted in Figure 7.

The most prominent observation in both confusion matrices is the dominance of the CONTAINS relation type. Especially, OVERLAP was often confused with CONTAINS in both datasets. This is not so surprising given that CONTAINS is a subset of OVERLAP. However, it seems that the stricter CONTAINS relation is used more often compared with the less restrictive OVERLAP relation. We assume that the reason for this phenomenon might be the fact that we hired medical students rather than linguists or computer scientists (an issue often observed in other annotation campaigns, as well). Thus, our annotators have attributed the relation types a more literal meaning, “something contains something”, even partially, and did not share our set theory-based understanding of CONTAINS and OVERLAP. Unfortunately, in the 3000PA_J_ corpus, all of the other relation types are often confused with the CONTAINS relation type. Discussions with the annotators revealed that they used the CONTAINS relation type as a residual category, which resulted in generally coding CONTAINS if they were unsure about other alternatives. However, mismatches between other labels occur very rarely (per mille range).

**Table 7:** Absolute numbers and relative numbers in percentage of temporal relations, mean pairwise F1-score with SD for each annotated type of the temporal relation annotation campaign on 3000PAJ and GraSCCo using temporal closure.

Relation type	3000PA_J_					GraSCCo			
	Absolute number	Percentage (%)	*F*_1_-score	SD	Absolute number		Percentage (%)	*F*_1_-score	SD
BEFORE	5948	10.07	0.2	0.07	547		9.43	0.45	0.28
BEGINS-ON	1030	1.74	0.48	0.07	146		2.52	0.64	0.19
ENDS-ON	724	1.23	0.69	0.06	74		1.28	0.73	0.14
OVERLAP	5046	8.54	0.09	0.13	679		11.71	0.33	0.36
CONTAINS	46,318	78.42	0.63	0.07	4352		75.06	0.68	0.16
Overall agreement	59,066	100.0	0.41	0.08	5798		100.0	0.57	0.27

### Baseline Model for Automatic Temporal Tagging

For the evaluation of the performance of the automatic temporal taggers trained on our annotated datasets, we fine-tuned and optimized a GBERT on both 3000PA_J_ and GraSCCo. A summary of the results of our experiments can be found in Table 8.

All in all, GBERT performs well for both the named entity recognition (NER) and the relation extraction task, respectively, on the 3000PA_J_ dataset. Training GBERT on 3000PA_J_ and testing on GraSCCo results in an expected, yet acceptable, drop in performance. Interestingly, this drop mostly affects the recall score. The union of both datasets results in a similar performance rate compared with the model trained on 3000PA_J_ only, yet with a slight decrease in recall and a slight increase in precision, which results in a similar *F*_1_-score for both the named entity recognition and the relation extraction task.

**Table 8:** Mean pairwise accuracy (Acc), F1-score, precision, and recall for automatic temporal named entity recognition (NER) and relation extraction (REX) on 3000PAJ and GraSCCo, respectively, and a unified set of both (union).

Model		Train set	Test set	Acc	*F*_1_-score	Precision	Recall
**NER**							
	GBERT^a^	3000PA_J_	3000PA_J_	0.95	0.78	0.73	0.84
	GBERT	GraSCCo	GraSCCo	0.94	0.85	0.84	0.87
	GBERT	3000PA_J_	GraSCCo	0.90	0.64	0.82	0.56
	GBERT	union	union	0.95	0.77	0.75	0.80
**REX**							
	GBERT	3000PA_J_	3000PA_J_	0.78	0.64	0.63	0.63
	GBERT	GraSCCo	GraSCCo	0.81	0.60	0.63	0.58
	GBERT	3000PA_J_	GraSCCo	0.90	0.64	0.82	0.56
	GBERT	union	union	0.81	0.63	0.70	0.59

^a^GBERT: German BERT model.

## Discussion

### Principal Findings

Temporal information is crucial for comprehensive medical reasoning; yet, only a few data resources are available for NLP tools to automatically extract such information from clinical documents. Publicly accessible clinical document collections have primarily emerged from challenge tasks, such as i2b2, n2c2, or Clinical TempEval, but cover the English clinical language only (Table 2). For a few non-English clinical language communities—French, Chinese (Mandarin), Spanish, Italian, Portuguese, German, Swedish, and Japanese—clinical corpora have been annotated with temporal metadata, but (except for E3C, a multilingual case report corpus) none of them is publicly available due to prohibitive data privacy regulations (Tables 4 and 8). We here introduced 2 corpora for the German clinical language annotated with TimeML-compliant metadata—a nondistributable real-world clinical corpus (3000PA_J_), and a distributable synthetic one (GraSCCo). The first stands out with unprecedented quantitative scales in terms of the number of documents, tokens, and temporal metadata, and its adherence to qualitative standards, that is, TimeML specifications, lacking for many non-English alternatives (Table 8). The latter, though small-sized and synthetic, is one of the rare (if not the only one) non-English clinical text resources marked up for temporal information that are accessible without distribution restrictions. With reasonable agreement scores for manual annotation, we trained and tested temporal BERT-based taggers under varying experimental conditions and achieved decent performance values using both, 3000PAJ and GraSCCo.

### Principal Results

In this article, we introduced 2 corpora equipped with temporal metadata for the German clinical language. The first of these corpora, 3000PA_J_, contains real clinical documents, but cannot be distributed in the public domain due to the restrictive privacy legislation codified in Germany and (more generally) Europe. To bypass this severe accessibility hurdle, we enriched a second corpus, GraSCCo, with temporal metadata, which can be distributed without any restrictions since it contains synthetic data, that is, fictitious and noise-adding content, only. The metadata we created for temporal named entities and relations, all of them fully compliant with TimeML, have a high level of credibility, given IAA scores ranging in the high 80s and low 90s.

As can also be seen from our evaluation results, we are on par performance-wise with comparable English corpora, such as THYME. For named entities and the CONTAINS relation, we even achieved better results on both corpora, 3000PA_J_ and GraSCCo. In addition, all documents used in the agreement evaluation were annotated by 4 individual annotators per document and yielded acceptable IAA scores.

In the end, we come up with about 66k temporal entities and 65k temporal relations, which sum up to 131k temporal annotation units for both corpora. These numbers not only exceed all clinical and nonclinical German-language corpora in terms of temporal metadata created up until now, but also outdo all clinical English time-focused corpora. Only the million-scale nonclinical Maven-Ere corpus [40] offers a (much) larger number of temporal annotations.

### Limitations

We still have to increase the size of GraSCCo (this is ongoing work in our research group). Also, the baseline tagger needs to be complemented by more sophisticated and larger language models. We plan to experiment with temporal (knowledge graph) embeddings, which encode temporal information directly in time-enhanced embeddings [99-101] and document-level event extraction models [102-104], which will better accommodate long-range cross-sentence and cross-paragraph dependencies typical of temporal relations in long documents, such as clinical reports.

As discussed in the results section, the decision to use only medical students resulted in misconceptions regarding the definitions of the temporal relations. Potentially, mixed teams with medical students and annotators with a background in linguistics or computer science might have resulted in better results (as small preliminary experiments for i2b2 2012 and CLEF suggest [49,105]). However, as shown by Raghavan et al [106], the level of clinical expertise has only a small impact on the inter-annotator reliability for temporal relations. Furthermore, medical students are more likely to recognize relations between clinical events [105], as well as relations based on medical causal dependence [105]. Additionally, we used the involuntary break and access restrictions during the COVID pandemic for extensive training iterations for our annotator team. Thus, if we compare our work with THYME, which was annotated by mixed teams as well, they reported similar difficulties and *F*_1_-scores to those discussed above.

### Comparison With Prior Work

To the best of our knowledge, 3000PA_J_ and GraSCCo are the first German clinical corpora to be annotated with temporal named entities and relations fully compliant with the TimeML de facto markup standard. Surprisingly, for nonclinical documents, no comprehensive TimeML-compliant corpus currently exists for German. Furthermore, providing unconstrained access to the synthetic GraSCCo corpus constitutes a major step in metadata availability for the German clinical NLP community. Moreover, the construction principles underlying GraSCCo [82] outline a way to extend the generation (not only) of temporal metadata beyond the German language. For the first time ever, a TimeML-compliant baseline temporal tagger for German has been trained and properly validated.

### Conclusions

Our work can and will be the basis for algorithms and models to automatically detect and combine temporal entities and their relations in order to create clinical timelines [107,108]. These timelines will be visualized and are thus able to provide a comprehensive summary of the patients’ clinical history, a potentially valuable source of abstraction for all kinds of clinical decision makers and caretakers.

## Appendix

Multimedia Appendix 1 Example for relation annotation schema.

Multimedia Appendix 2 Hyperparameter for transformer training.

## Abbreviations

BERT: Bidirectional Encoder Representations from Transformers
GBERT: German BERT model
IAA: interannotator agreement
ISO: International Organization for Standardization
NLP: natural language processing
