# Supplementary material for: Temporal Annotation of German Clinical Language in Real and Synthetic Clinical Documents: Corpus Development and Baseline Tagger Validation Study
Source: J Med Internet Res. 2026 Feb 25;28:e71458. doi: 10.2196/71458 (PMC12980054; doi:10.2196/71458)
Supplement: Multimedia Appendix 1 [file jmir_v28i1e71458_app1.pdf]

Schema for Relation Annotation

1. Named Entity Annotation (simplified)

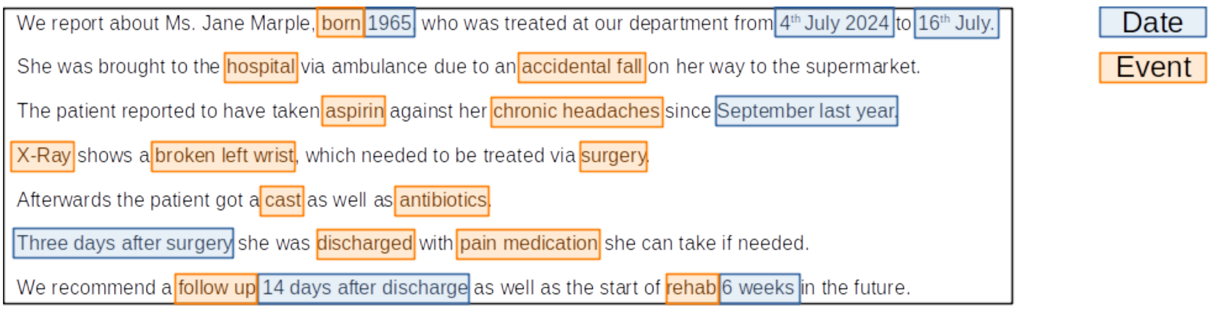

This is a short example of a fictional hospital stay that has been annotated with TIMEX3 and EVENT named entities.

2. First connection step

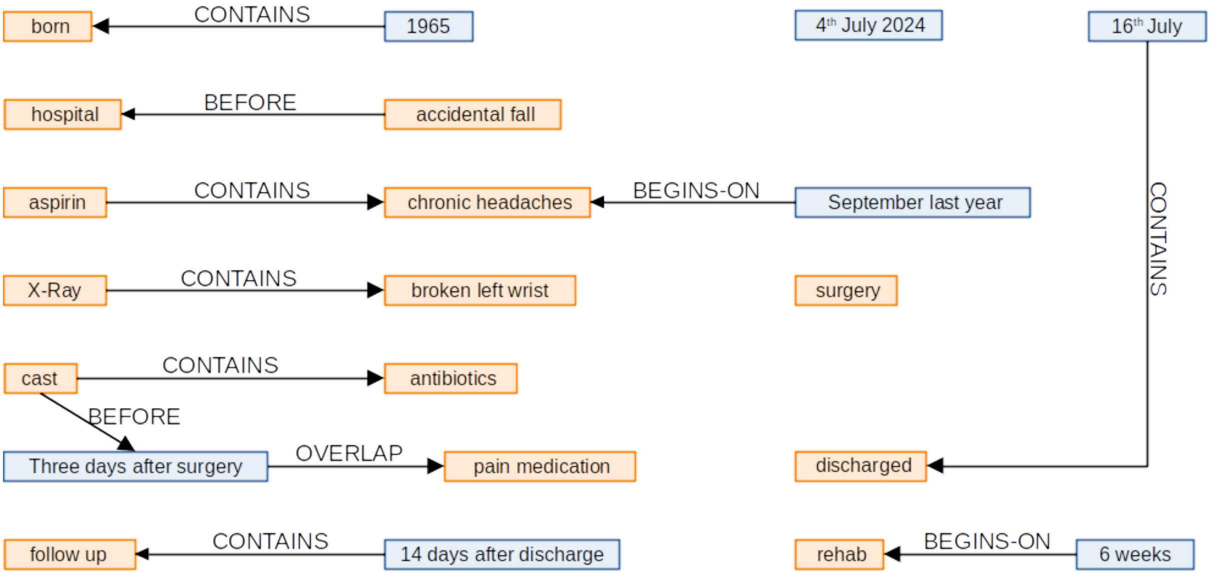

First, the annotators were encouraged to only connect the most closely related entities. In this example we eliminated the text for a better overview, but kept the positions in the sentences.

3. Iterative connection step(s)

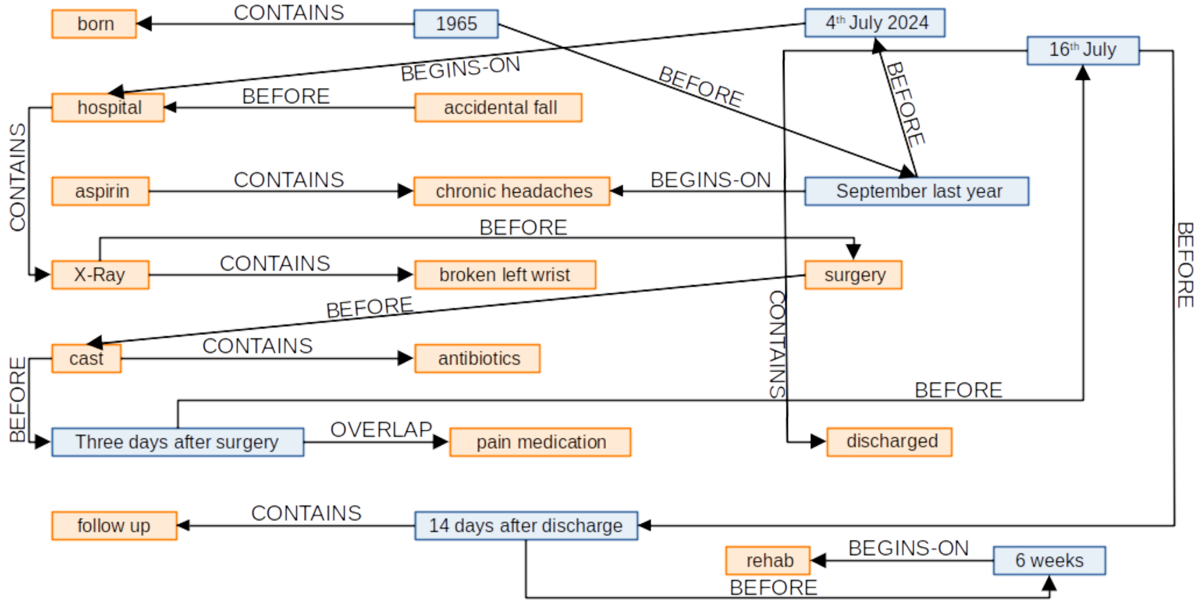

The goal is to connect these smaller units of meaning in a way that every unit has a connection to all other units. Some units are capsuled, thus more than one iteration is needed. This way we have one temporal graph with as few connections as possible. A minimum spanning tree would be ideal but was not forced.

These units of meaning all need some entity as an anchor point to connect with other units of meaning or entities. Here, TIMEX3 entities are preferred.

4. Final (sorted) Timeline

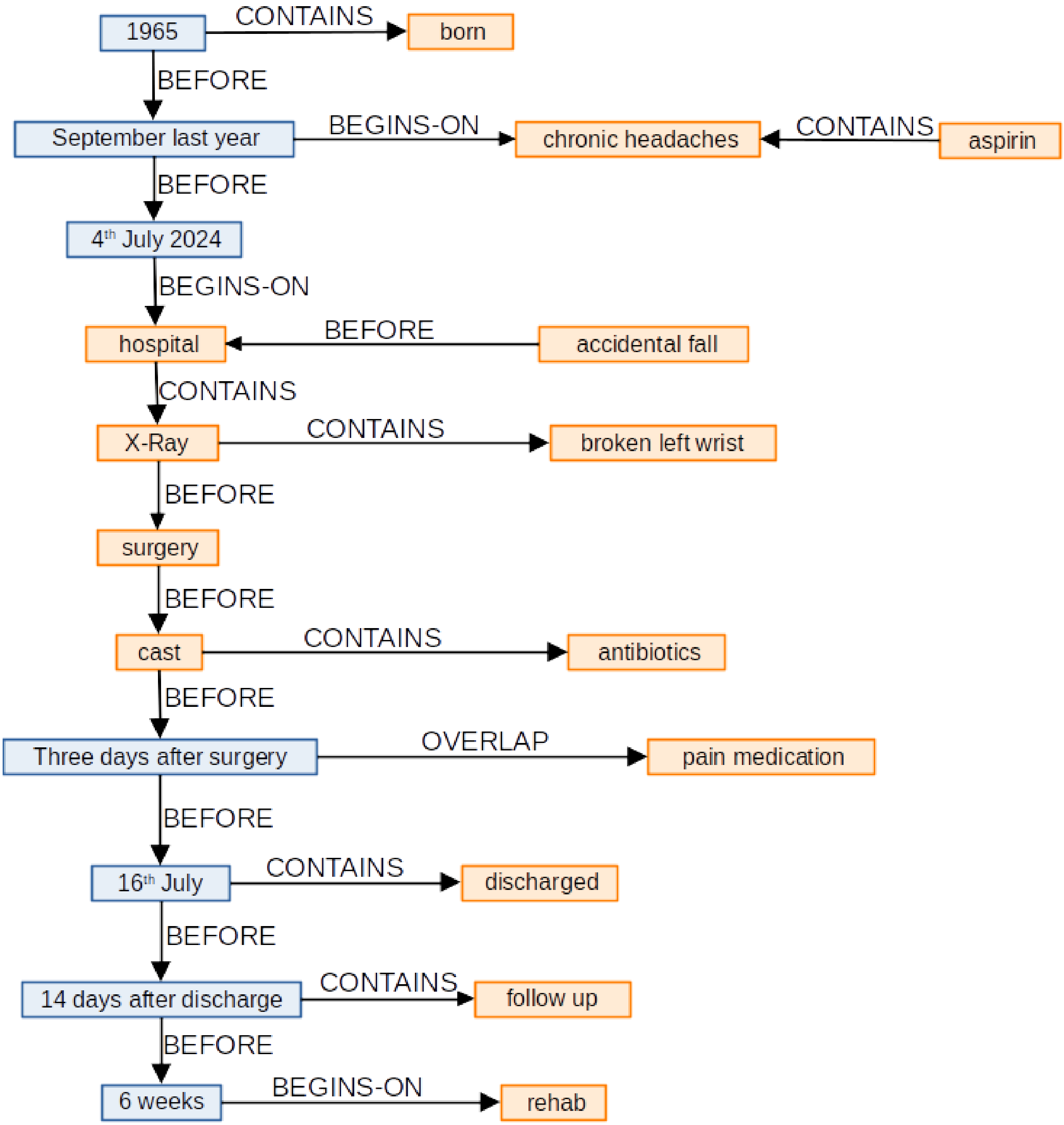

This timeline is for demonstration purposes and is not part of the annotation process. It can be generated automatically from the annotated temporal relation information
