# Supplementary material for: Temporal Annotation of German Clinical Language in Real and Synthetic Clinical Documents: Corpus Development and Baseline Tagger Validation Study
Source: J Med Internet Res. 2026 Feb 25;28:e71458. doi: 10.2196/71458 (PMC12980054; doi:10.2196/71458)
Supplement: Multimedia Appendix 2 [file jmir_v28i1e71458_app2.docx]

# Hyperparameter for Baseline Training

## Named Entity Recognition

### Fixed Parameters:

- Model: deepset/gbert-base
- Evaluation Metric: F1-Score
- Training Epochs: 100

| Train data | Test data | Batch Size | Gradient Accumulation steps | Learning Rate | Maximum sequence length |
| --- | --- | --- | --- | --- | --- |
| 3000PA_J_ | 3000PA_J_ | 2 | 1 | 0.00005 | 400 |
| GraSCCo | GraSCCo | 8 | 4 | 0.0005 | 350 |
| 3000PA_J_ | GraSCCo | 4 | 2 | 0.000005 | 500 |
| union | union | 2 | 1 | 0.00005 | 350 |

## Relation Extraction

### Fixed Parameters:

- Model: deepset/gbert-base
- Evaluation Metric: F1-Score
- Training Epochs: 10
- Context window size (number of token left and right of the named entity pairs): 200

| Train data | Test data | Batch Size | Gradient accumulation steps | Learning Rate | Padding to max. length | Group by length | Maximum sequence length |
| --- | --- | --- | --- | --- | --- | --- | --- |
| 3000PA_J_ | 3000PA_J_ | 8 | 2 | 0.00005 | true | true | 350 |
| GraSCCo | GraSCCo | 4 | 2 | 0.00005 | false | true | 400 |
| 3000PA_J_ | GraSCCo | 4 | 2 | 0.000005 | true | false | 450 |
| union | union | 4 | 2 | 0.000005 | false | false | 400 |
